# First full-genome alignment representative for the genus *Pestivirus*

**DOI:** 10.1261/rna.080732.125

**Published:** 2026-03

**Authors:** Sandra Triebel, Tom Eulenfeld, Nancy Ontiveros-Palacios, Blake Sweeney, Norbert Tautz, Manja Marz

**Affiliations:** 1 RNA Bioinformatics and High-Throughput Analysis, Friedrich Schiller University Jena, 07743 Jena, Germany; 2 European Virus Bioinformatics Center, Friedrich Schiller University Jena, 07743 Jena, Germany; 3 European Molecular Biology Laboratory, Wellcome Genome Campus, European Bioinformatics Institute, Hinxton, Cambridge CB10 1SD, United Kingdom; 4 Institute of Virology and Cell Biology, University of Luebeck, 23562 Luebeck, Germany; 5 Fritz Lipmann Institute-Leibniz Institute on Aging, 07745 Jena, Germany

**Keywords:** *Pestivirus*, full-genome alignment, RNA secondary structures

## Abstract

Members of the genus Pestivirus (family *Flaviviridae*) comprise economically important livestock pathogens like classical swine fever virus (CSFV) and bovine viral diarrhea virus (BVDV). Research over recent years revealed 11 recognized and eight proposed species. The single-stranded, positive-sense RNA genome encodes one large polyprotein that is processed by viral and cellular proteases into 12 mature proteins. In addition to its protein-coding function, the RNA genome contains secondary structures critical for various stages of the viral life cycle. Some of these structures, including the internal ribosome entry site (IRES) and a 3′ stem–loop, essential for genome replication, have been studied in individual pestiviruses. Here, we present the first genome-wide multiple sequence alignment comprising all known pestivirus species (accepted and tentative) and a comprehensive analysis of phylogenetically conserved RNA secondary structures across the genus. Well-characterized elements, such as a 5′ stem–loop, the IRES, and the 3′ stem–loop SL I, were conserved in all pestiviruses, whereas additional 3′ untranslated region structures were conserved only in subsets of species. We identified 29 novel conserved RNA secondary structures within the protein-coding region, with thus far unresolved functional importance. A miR-17 binding site, previously described in species A, B, and C, was detected in ten additional species but absent in species K, S, Q, and R. We identified a putative long-distance RNA interaction between the IRES and the 3′ end of the genome. Together, these findings and the comprehensive MSA of all 19 pestivirus species provide a valuable resource for future research and diagnostic applications.

## INTRODUCTION

The members of the genus *Pestivirus* (family *Flaviviridae*) are enveloped, positive-strand RNA viruses with genome lengths ranging from ∼11.6 to 12.3 kb (Tautz et al. 2015; Mifsud et al. 2024). Currently, the ICTV lists 11 pestivirus species, and eight more have been proposed (Table 1; Postel et al. 2021). Pestiviruses like the bovine viral diarrhea viruses (BVDV-1 and BVDV-2) and classical swine fever virus (CSFV) are long-known animal pathogens with high relevance to stock farming worldwide (Tautz et al. 2015). Formerly, pestiviruses were thought to be restricted to cloven-hoofed animals as their hosts. However, when major improvements in bulk sequencing uncovered the sequences of rat pestivirus, *Phocoena* pestivirus (PhoPeV) from harbor porpoises, and pangolin pestivirus, as well as the atypical pestiviruses from bats, it became evident that members of this genus infect a much broader host spectrum (Postel et al. 2021). Recently, even more distantly related pestiviruses were identified by protein structure prediction and comparison combined with RNA sequence analysis (Mifsud et al. 2024).

**TABLE 1.** Overview of *Pestivirus* species and corresponding host species

Species	Species abbr.	Virus name	Abbr.	Host species
*Pestivirus bovis*	A	Bovine viral diarrhea virus 1	BVDV-1	*Bos* spp., *Ovis* spp., *Capra* spp., *Artiodactyla*
*P. tauri*	B	Bovine viral diarrhea virus 2	BVDV-2	*Bos* spp., *Ovis* spp., *Capra* spp., *Artiodactyla*
*P. suis*	C	Classical swine fever virus	CSFV	*Sus scrofa*
*P. ovis*	D	Border disease virus	BDV	*Ovis* spp., *Capra* spp., *Artiodactyla*
*P. antilocaprae*	E	Pronghorn antelope pestivirus	PAPeV	*Antilocapra americana*
*P. australiaense*	F	Porcine pestivirus	PPeV	*S. scrofa*
*P. giraffae*	G	Giraffe pestivirus	GPeV	*Bos taurus*, *Giraffa camelopardalis*
*P. brazilense*	H	HoBi-like pestivirus	HoBiPeV	*Bos* spp.
*P. aydinense*	I	Aydin-like pestivirus	AydinPeV	*B. taurus*, *Ovis* spp., *Capra* spp.
*P. ratti*	J	Rat pestivirus	RPeV	*Rattus norvegicus*
*P. scrofae*	K	Atypical porcine pestivirus	APPeV	*S. scrofa*
*Pestivirus L* ^*^	L	Linda virus	LindaV	*S. scrofa*
*Pestivirus M* ^*^	M	*Phocoena* pestivirus	PhoPeV	*Phocoena phocoena*
*Pestivirus N* ^*^	N	Tunisian sheep-like pestivirus	TSV	*Capra aegagrus hircus*, *Ovis aries*
*Pestivirus O* ^*^	O	Ovine/IT pestivirus	ovIT-PeV	*O. aries*
*Pestivirus P* ^*^	P	Pangolin pestivirus	DYPV	*Amblyomma javanense*, *Manis javanica*
*Pestivirus Q* ^*^	Q	Rodent pestivirus	RtNn-PeV	*Niviventer niviventer*
*Pestivirus R* ^*^	R	Rodent pestivirus	RtAp-PeV	*Apodemus peninsulae*
*Pestivirus S* ^*^	S	Bat pestivirus	BtSk-PeV	*Scotophilus kuhlii*
*Pestivirus*		Wenzhou (YJB_Pabr)		*Pipistrellus abramus*
*Pestivirus*		Zikole		*B. taurus* (metagenome)
*Pestivirus*		Jingmen (SYS_SheQu)		*Crocidura shantungensis*
Pesti-like viruses		ZJU-Q2		*Bemisia tabaci*
Pesti-like viruses		Trinbago (TTP-Pool-4)		*Rhipicephalus sanguineus*
Pesti-like viruses		CT2		*Dermacentor reticulatus*

Eleven species are already known (A–K), and eight new species (L–S) highlighted with “*” are proposed by Postel et al. (2021). Three entries are not assigned to any species but were reported as pestiviruses in our data set. Three genomes were reported as Pesti-like viruses.

The pestiviral genome is organized into a single open reading frame (ORF) flanked by two untranslated regions (UTRs) at the 5′ and 3′ ends. The ORF encodes the polyprotein NH_2_-*N^pro^-C-E^rns^-E1-E2-p7-NS2-NS3-NS4A-NS4B-NS5A-NS5B*-COO-, with the exception of PhoPeV, which does not encode for the N-terminal protease *N*^*pro*^ (Jo et al. 2019). The polyprotein is processed by ER-resident cellular protease as well as the viral proteases *N*^*pro*^, nonstructural protein 2 (*NS2*), and *NS3/4A* into 12 proteins (Tautz et al. 2015). *N*^*pro*^ and the envelope glycoprotein ribonuclease secreted (*E*^*rns*^) are found only in pestiviruses and are crucial for combating the host's innate immune response (Bauhofer et al. 2007; de Martin and Schweizer 2022). The cleavage of *NS2–3* into *NS2* and *NS3* by the cysteine protease in *NS2* is vital for pestiviral RNA replication and depends on *NS2* protease activation by the cellular chaperone DNAJC14 (formerly termed Jiv) (Rinck et al. 2001). For *NS2* protease activation, the Jiv90 domain of DNAJC14 is sufficient (Rinck et al. 2001). Another peculiarity of pestiviruses is the generation of virus mutants by RNA recombination during persistent infection of ruminants. The cell-derived sequences in the viral genomes encode, for example, DNAJC14, ubiquitin (Ub), SUMO, LC3, or NEDD3 and are causative for a change in the viral biotype and the induction of lethal disease in the persistently infected host (Brownlie et al. 1984; Becher and Tautz 2011).

The pestivirus genome carries neither a 5′ cap nor a 3′ poly(A) tail. Initiation of translation of the viral RNA genome is mediated by a 5′ internal ribosome entry site (IRES), a complex RNA structure that recruits the ribosomes directly to the start codon of the single large open reading frame (ORF) (Poole et al. 1995; Gosavi et al. 2022). The pestiviral IRES belongs to type IV, analogous to the one in the hepatitis C virus (HCV) genome (Arhab et al. 2020). Further RNA structures at the 5′ and 3′ ends are crucial for recruiting the viral replicase to the RNA for genome amplification. In the 3′ noncoding region, three stem–loop (SL) structures have been described with the 3′-terminal one, SL I, being of the highest functional relevance (Yu et al. 2000; Isken et al. 2004; Pankraz et al. 2005). More recently, upstream of SL I highly conserved let-7 and miR17 binding sites were identified (Scheel et al. 2017); miR17 binding was shown essential for replication of BVDV-1 (Kokkonos et al. 2020).

Here we present the first full-genome alignment of pestiviruses coupled with RNA secondary structure annotation. The alignment was generated using a semiautomated approach and curated by experts in the field of the genus *Pestivirus* and its associated structural elements. In addition to established structures such as the 5′ UTR IRES and 3′ UTR SL I, our study reveals previously unrecognized RNA secondary structures, especially in the protein-coding region, which were predicted by computational methods and thus are conserved in all pestivirus genomes. The results of our alignment—using genomes covering the entire phylogenetic sequence space of pestivirus isolates—suggest that our predictions are likely to cover virtually all possible RNA secondary structures conserved for all known pestiviruses.

## RESULTS AND DISCUSSION

**FIGURE 1.**
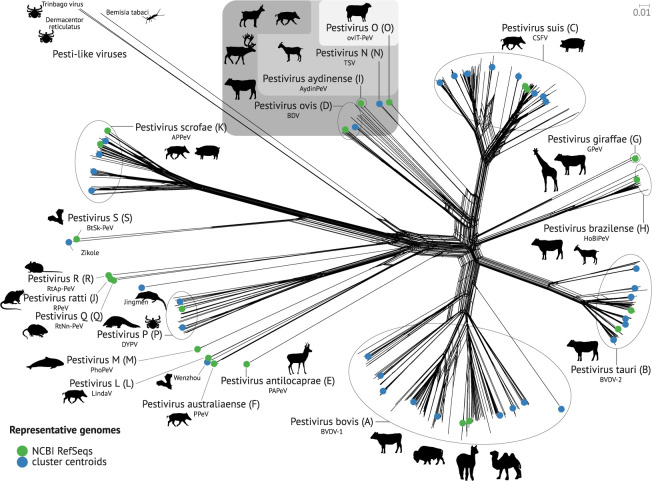
Phylogenetic representation of the *Pestivirus* data set. Our representative genomes consist of NCBI RefSeq genomes (green) and cluster centroids (blue). The tree shows a clear distinction between the different species. The hosts reported for the species are shown by the pictograms. Table 1 provides a detailed overview of the hosts. The tree is based on the MAFFT alignment of all complete-genome sequences and served as input for SplitsTree to construct a split graph using the neighbor-net algorithm.

**FIGURE 2.**
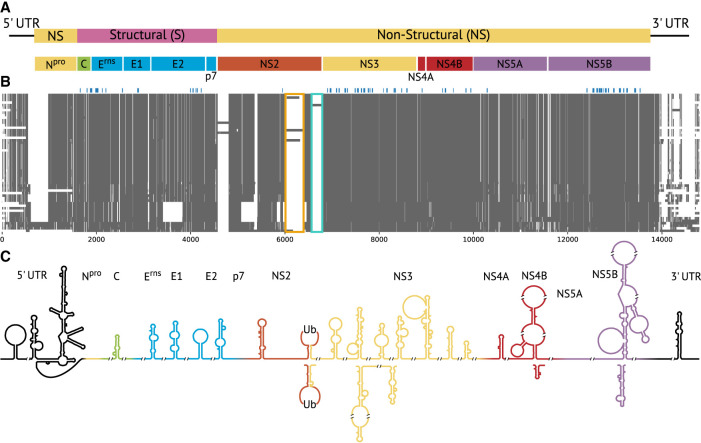
Overview of the (*A*) genome organization (adapted from ICTV) of *Pestivirus*, (*B*) full-genome alignment, and (*C*) RNA secondary structures. In *B*, gray areas display the sequences, and white areas represent gap regions. Blue above the alignment highlights the location of the anchors calculated by AnchoRNA. Colored boxes highlight the insertion of DNAJC14 (orange) and ubiquitin (Ub, cyan). In *C*, RNA secondary structures colored in black are already described in the literature; colored ones are novel structures derived from the in silico predictions in this study.

In this study, we present a comprehensive analysis of all conserved RNA secondary structures that occur in the complete sequence space of all downloadable pestivirus genomes (Fig. 1). Thus, the results aim to provide a complete description of RNA secondary structures that may be advantageous for the viral life cycle; see overview in Figure 2.

We found 32 conserved RNA secondary structures present in all pestiviruses as well as six selected RNA secondary structures present only in subsets of pestiviruses.

For pestiviruses, functionally important RNA secondary structures in the untranslated regions are known. However, with the notable exception of a computational prediction (Thurner et al. 2004), no RNA secondary structures in coding regions have been described thus far. A variety of molecular mechanisms can be envisioned in which RNA secondary structure elements are involved. Compared to other *Flaviviridae* RNA secondary structure elements in the protein-coding region, we can imagine the influence on the translation outcome of a particular RNA (Firth and Brierley 2012), for example, by inducing ribosome frameshifts or termination reinitiation. Specific RNA elements can be used to selectively package one RNA, while another, longer RNA species is excluded from packaging by translational inactivation (Nassal et al. 1990). A translating ribosome may also displace proteins from a secondary structure element of the RNA and thereby initiate a kind of “burn after reading” degradation of the RNA (Chang et al. 2004). Therefore, it is important to identify those RNA secondary structures that have been selected for their function from the available sequence space produced by the error-prone replicases of RNA-plus-strand viruses.

### Basic statistics of the full-genome alignments

Our nucleotide-based alignment contains 55 representative *Pestivirus* genomes covering all 19 species (*Pestivirus* A–S). The alignment spans 14,793 residues and 143,147 gaps, averaging ∼2603 gaps per sequence. Only four sequences within our alignment contain a total of 11 positions with non-ACGU characters (R, M, Y, S, K, and N; see IUPAC code).

**FIGURE 3.**
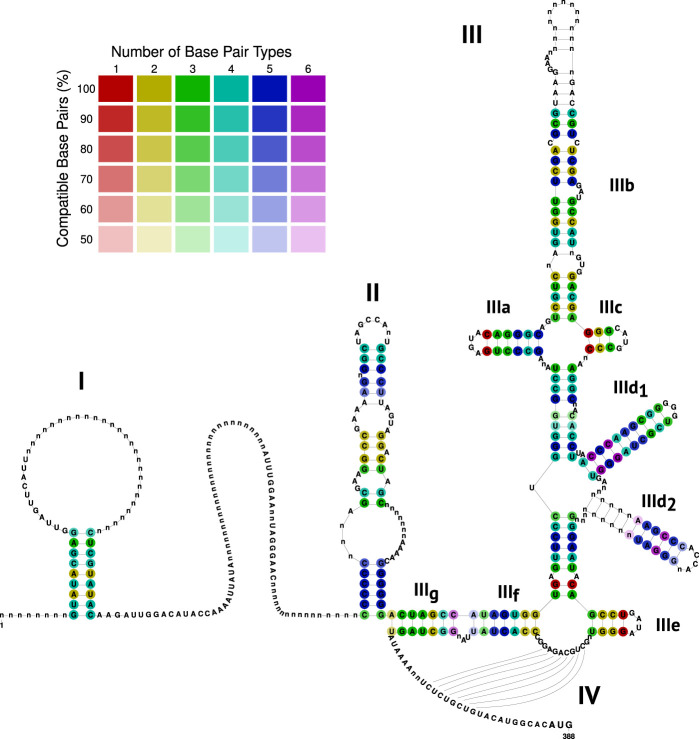
Alignment-based RNA secondary structure prediction of the 5′ UTR, colored by the number of base pair types and the percentage of sequences that form the base pairs, illustrating the extent of covariations in double-stranded regions. Among the selected sequences, 51 isolates had a 5′ UTR sequence (32 complete, 19 incomplete). The 5′ UTR contains D I (present in 32 sequences) and the IRES, which comprises the domains D II (present in 46 sequences), D III, and D IV (both present in 48 sequences) upstream of the polyprotein start codon.

The full 5′ UTR exists for 32 sequences (19 incomplete); three sequences lack the 5′ UTR entirely, and we manually removed the 5′ UTR sequence of the Jingmen strain (isolate SYS_SheQu, accID OM030319) due to no sequence or structure similarity. Additional information about the Jingmen strain is provided in the Supplemental Material. On a structural level, 32 sequences contain domain I (D I), 46 sequences D II, and 48 sequences D III and IV. Domains II, III, and IV form the IRES (Fig. 3). For the 3′ UTR, 38 sequences display the complete 3′ UTR; three sequences completely lack the 3′ UTR. Only SL I is conserved across all species.

We added additional information to our nucleotide alignment: (1) gene annotations (shown as annotation line #=GC Annotation in the stk file); (2) RNA secondary structures (including pseudoknots) documented in the literature (lines #=GC SS_cons, #=GC SS_cons_PK and the corresponding names specified as roman numerals in #=GC SS_cons_names); and (3) in silico predicted novel RNA secondary structures (including alternative conformations), providing a comprehensive view of potential conformations within the *Pestivirus* genome (lines #=GC SS_cons, #=GC SS_cons_alternative and corresponding names in #=GC SS_cons_names, #=GC SS_cons_names_alternative denoted as “SL” and start position in the genome 1-SD1, accID NC_076029) (Table 2; Supplemental File F6).

**TABLE 2.** Conserved RNA secondary structures (SS) in *Pestivirus* genome, along with their corresponding Rfam model IDs (Griffiths-Jones et al. 2003; Ontiveros-Palacios et al. 2025)

RNA SS	Genomic region	Alignment position		1-SD1 position	
		From	To	From	To
SL I	5′ UTR	9	64	1	32
IRES (RF00209)	5′ UTR	153	481	77	361
**SL 1003**	*C*	1570	1605	1003	1035
**SL 1389**	*E* ^*rns*^	1983	2033	1389	1439
**SL 2028**	*E1*	2649	2706	2028	2085
**SL 3079**	*E2*	3763	3808	3079	3124
**SL 3385**	*E2*	4090	4151	3385	3446
**SL 5050**	*NS2*	6463	6522	5050	5109
**SL 5096**	*NS2/3*	6509	6921	5096	5280
**SL 5138**	*NS2/3*	6551	6802	5138	5161
**SL 5255**	*NS3*	6896	6962	5255	5321
**SL 5302**	*NS3*	6943	7260	5302	5619
**SL 5450**	*NS3*	7091	7113	5450	5472
**SL 5453**	*NS3*	7094	7189	5453	5548
**SL 5807**	*NS3*	7451	7506	5807	5862
**SL 5898**	*NS3*	7542	7611	5898	5967
**SL 6000**	*NS3*	7644	7701	6000	6057
**SL 6021**	*NS3*	7665	7812	6021	6168
**SL 6602**	*NS3*	8249	8294	6602	6647
**SL 6795**	*NS3*	8445	8471	6795	6821
**SL 7217**	*NS4A*	8879	8912	7217	7250
**SL 7796**	*NS4B*	9458	9849	7796	8187
**SL 7805**	*NS4B*	9467	9498	7805	7836
**SL 7900**	*NS4B*	9562	9768	7900	8106
**SL 8102**	*NS4B*	9764	9784	8102	8122
**SL 10714**	*NS5B*	12,550	13,439	10,714	11,600
**SL 10805**	*NS5B*	12,641	13,113	10,805	11,277
**SL 10828**	*NS5B*	12,664	12,706	10,828	10,870
**SL 10951**	*NS5B*	12,787	12,827	10,951	10,991
**SL 11497**	*NS5B*	13,336	13,402	11,497	11,563
**SL 11553**	*NS5B*	13,392	13,456	11,553	11,617
SL I (RF04322)	3′ UTR	14,716	14,782	12,249	12,304

Start and end positions refer to the alignment and reference strain 1-SD1. We updated the IRES Rfam model RF00209, confirmed the previously predicted RNA secondary structure of SL I in the 3′ UTR in Rfam model RF04322 (available in Rfam v15.1 or later), and predicted a further 29 novel conserved RNA families (bold font), which will be incorporated into Rfam. We named novel structures according to their start position in the genome 1-SD1 (accID NC_076029).

The protein alignment of the 55 representative genomes encompasses a total of 4480 residues, with an average of 608 gaps (33,433 gaps in total). We provide the nucleotide and protein alignments in the Supplemental Material with several formats, such as Stockholm (stk), ClustalW (aln), and Fasta (fasta) (Supplemental Files F6–F8), which can be conveniently visualized using tools such as ClustalX (Jeanmougin et al. 1998; Larkin et al. 2007), Jalview (Clamp et al. 2004), or Emacs RALEE mode (Griffiths-Jones 2005).

### The 5′ UTR of pestiviruses

The 5′ UTR of pestiviruses consists of 5′ to 3′ end of domain I (D I), a variable region, domain II (D II), and domain III (D III), which is divided into SL IIIa, IIIb, IIIc, IIId__1__, IIId__2__, IIIe, IIIf (also known as stem 1b), and IIIg (also known as stem 1a) (Fig. 3). A pseudoknot (PK, IV) is formed by pairing the region between IIIe and IIIf with the region directly upstream of the start codon (Fig. 3). D II, D III, and the pseudoknot form together the internal ribosomal entry site (IRES). The alignment of the 5′ UTR is stored in Supplemental File F9.

#### D I and D II

The very 5′-terminal stem–loop structure of BVDV 5′ UTR has been represented as a bifunctional RNA signal involved in translation and RNA replication (Yu et al. 2000). We could identify this domain to be present in all *Pestivirus* species with some expansions of the hairpin loop region; it can be up to 42 nt in length (NC_077024, species P).

The variable region between D I and D II harbors for most species, a stable hairpin, which is however not at a fixed location but “moves” from species to species up/downstream, as shown previously for D42109 and D42110 (both species C) (Katayama et al. 1995). The D I hairpin also varies in length, shape, internal, and hairpin loops (Supplemental Fig. S2). Three sequences of species C, one of D, and one each of P and Q are predicted to form two hairpins. Some sequences of species A, C, K, and the Zikole strain cannot form a hairpin between D I and D II. Interestingly, despite all the variant RNA secondary structures, the primary sequence is relatively conserved (Supplemental Fig. S3).

The hairpin loop always contains the sequence CCA, and the 3′ part of the stem consists of the sequence GUA in an internal loop in all *Pestivirus* species, suggesting a possible protein interaction (Supplemental Fig. S4). Domain II was suggested by Gosavi et al. to be Y-shaped and supported by SHAPE data in BVDV-1. We were able to show a potential Y-shaped D II alternative structure for most *Pestivirus* species; however, species P, K, and S cannot form a Y-shape. Since these three species are phylogenetically not closely related, we reject a general Y-shape structure of D II in pestiviruses.

#### IRES

The IRES of pestiviruses has been analyzed for many years and been well described for decades (Brown et al. 1992; Deng and Brock 1993; Katayama et al. 1995) in BVDV (species A and B) and hog cholera virus (HoCV, nowadays known as classical swine fever virus—CSFV, species C). However, although the Rfam DB contains species A–D and G of the IRES, a comparison of species D–S has not yet been performed.

All species share all elements of the IRES, although the sequences differ. However, the RNA secondary structures vary only slightly in the shape and length of the individual hairpins of the IRES. In this study, the IRES element could be computationally retrieved without manual curation with the described methods. Stem IIIg (7 nt) and IIIf (7 nt with one internal loop of 1 nt) are well conserved and completely match in species B, E, and O. Species A, C, D, F, I, L, M, and N show a single base pair that does not form, which presumably does not affect the stability of the stem. However, species P, K, Q, R, and J have a disruptive stem IIIg and stem IIIf, which results in a slightly less stable RNA–RNA interaction (ΔMFE = 1.5 kcal/mol). Stem–loop IIIa has in each species exactly the length of 6 nt with a hairpin loop length of 4 nt, indicating the evolutionary constraints of that hairpin. The strongly conserved consensus motif for the hairpin loop and closing base pair is GAGUAC. Hairpin IIIb varies in length (16–25 nt) and stability (MFE ranging from −13.50 to −27.60 kcal/mol). The hairpin loops of species K and S are longer (25 nt compared to usually 19 nt). Stem IIIc is highly compensatory mutated, but maintains in each species the same length of 3 nt, with a variant hairpin loop of 3 (species Q, R, and J) or 4 nt (other species), which is a conserved motif in species A, B, and C, but divergent for other species. Stem IIId__1__ is in each species exactly 10 nt long with a hairpin loop of 4 or 5 nt always containing the sequence GGGG. Stem IIId__2__ differs from species to species in sequence, length (3–11 nt), and hairpin length. Stem IIIe consists of 4 bp (or 3 bp in some variants of species C) and 4 nt in the loop region.

#### Pseudoknot

**FIGURE 4.**
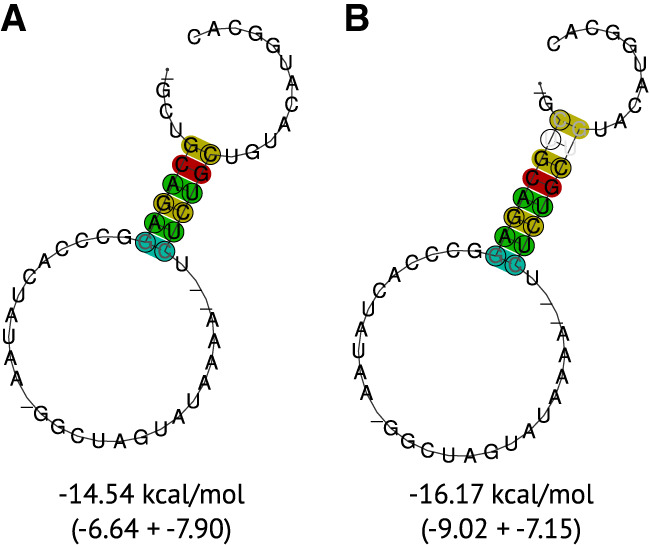
Consensus RNA secondary structure of the pseudoknot in the 5′ UTR of the (*A*) 6 nt stem–loop and (*B*) 8 nt stem–loop. The structures were predicted and visualized using RNAfold with additional parameters for structural constraints. The constraints forced the lengths of the stem–loops. The averaged MFE in kcal/mol (composed of actual MFE and covariance pseudo-energy) is displayed below the structure.

The IRES pseudoknot is very stable in each species (MFE ranging from −6.20 to −10.60 kcal/mol). Some publications suggest an additional stem–loop with 2 bp between IIIe and IIIf, in which the hairpin serves as part of the pseudoknot (Brown et al. 1992; Fletcher and Jackson 2002). However, this cannot be confirmed for most of the species and has recently also been shown by SHAPE data not to be present for BVDV (Gosavi et al. 2022). We propose instead an extended, slightly more stable pseudoknot interaction of 8 nt instead of only 6 nt, as also previously suggested by SHAPE data (Gosavi et al. 2022); see Figure 4 (ΔMFE = 1.63 kcal/mol).

Another hairpin has been discussed several times directly upstream of the start codon (Fletcher and Jackson 2002). We could not find this hairpin conserved for all pestiviruses. We hypothesize this hairpin to be present for all pestivirus species A, B, and C, as well as in some other pestivirus isolates (G, H, D, I, N, O, and E). However, its functionality remains debated, because this hairpin competes with the formation of the pseudoknot.

### The 3′ UTR of pestiviruses

**FIGURE 5.**
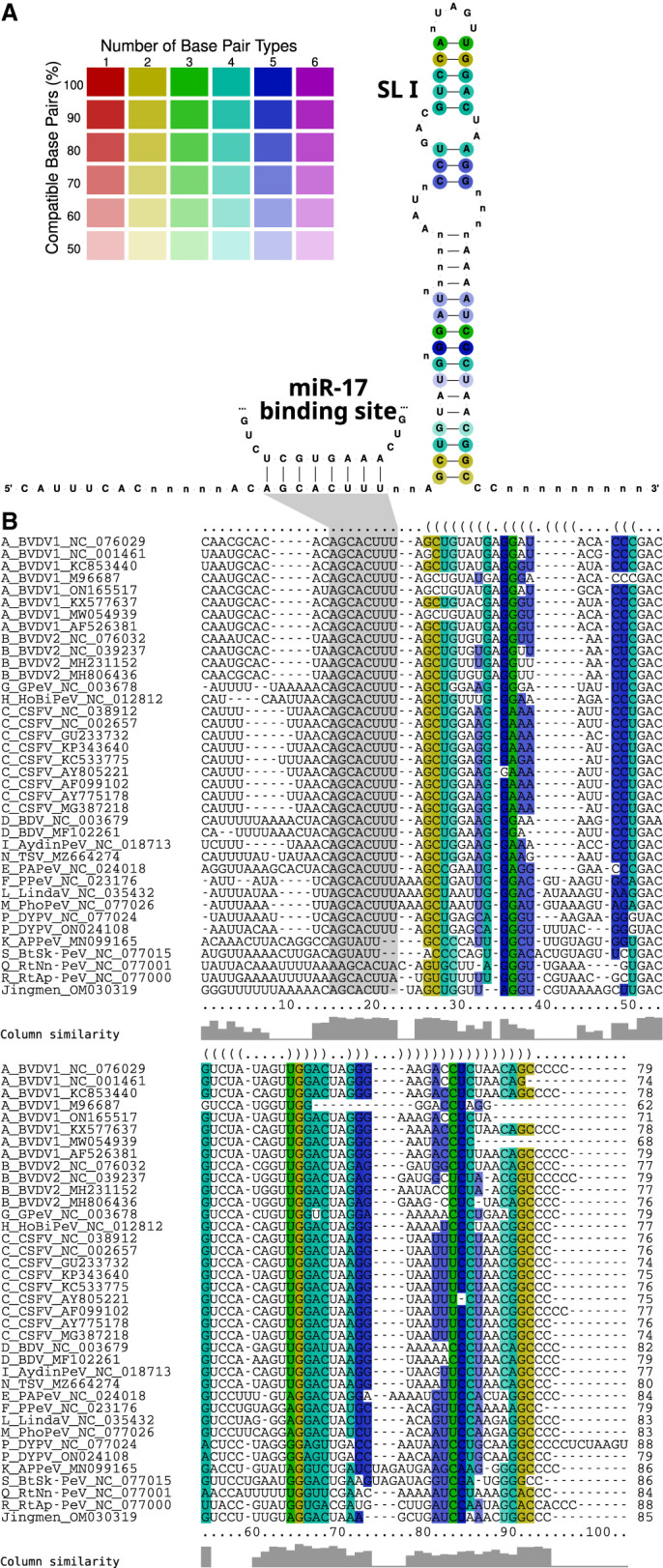
(*A*) RNA secondary structure of the 3′ UTR SL I, colored by the number of base pair types and the percentage of sequences that form the base pairs, illustrating the extent of covariations in double-stranded regions. (*B*) Multiple sequence alignment of the 3′ UTR SL I. Among the selected sequences, 38 isolates had a 3′ UTR sequence in the region of SL I. The binding site for miR-17 is located upstream of the stem–loop with the seed sequence AGCACUUU present in 33 sequences (highlighted in gray).

The 3′ UTR of pestiviruses has been shown mainly for BVDV and CSFV to consist of stem–loops (SL) IV, III, II, and I (Deng and Brock 1993; Yu et al. 1999). A comprehensive analysis of the 3′ UTR across several hundred *Pestivirus* sequences has not yet been performed. Within our analysis, only SL I is conserved across all *Pestivirus* species (Fig. 5; Supplemental File F10). Although SL I shows sequence divergence outside of pestivirus species A, B, and C, it still forms a conserved secondary structure, as shown in Supplemental Figure S6. According to a study by Pankraz et al. (2005), the presence of SL I and the 3′-terminal region between SL I and II are crucial for pestivirus replication. In contrast, deletions within SL II and SL III, or even the absence of either of these loops, did not significantly affect viral replication. However, a mutant RNA lacking both SL II and SL III was found to be noninfectious. This suggests that while SL II and SL III are less critical individually, the presence of at least one of these elements is most likely to be essential for the replication of the BVDV-1 strain CP7.

#### MicroRNA binding sites

MiR-17 is known to bind to the 3′ UTR of pestiviruses in species A, B, and C (Scheel et al. 2016; Kokkonos et al. 2020). The binding of miR-17 to the 3′ UTR is essential for replication, enhances viral translation, and increases RNA stability. In the present work, the seed sequence (AGCACUUU) of the miR-17 binding site directly upstream of SL I is shown to be perfectly conserved in 33 sequences from 13 species covering that region. Species K, S, Q, R, and the Jingmen strain show mutations and deletions in the seed sequence (Fig. 5), thus implying that no interaction with miR-17 takes place. No sequences of species O and J were available for that region.

The binding of microRNA let-7 is known to increase viral translation to its full efficiency (Kokkonos et al. 2020). In species A and C, the binding site is located in the hairpin loop of SL II. The seed sequence CUACCUCA is located further upstream of the miR-17 binding site. Here, we identified 38 representative sequences from 11 species (A–D, F–I, L, N, and O) that show a strong conserved binding site of let-7.

#### Species-specific structures and conserved regions

Further structures in the 3′ UTR (SL II, SL III, SL IV) were reported for the species A–C (Deng and Brock 1993; Isken et al. 2004; Pankraz et al. 2005). However, the 3′ UTR region is very heterogeneous (Thurner et al. 2004), as the 3′ end of S IV in species A appears homologous to the 5′ end of S III in species C.

Moreover, SL II appears larger in species A than in other species. Also, SL III can be formed only for longer sequences (NC_076029, NC_001461, ON165517) of species A, as previously reported (Deng and Brock 1993; Isken et al. 2004). Species-specific alignments for the 3′ UTR are stored in Supplemental Files F11–F13.

**FIGURE 6.**
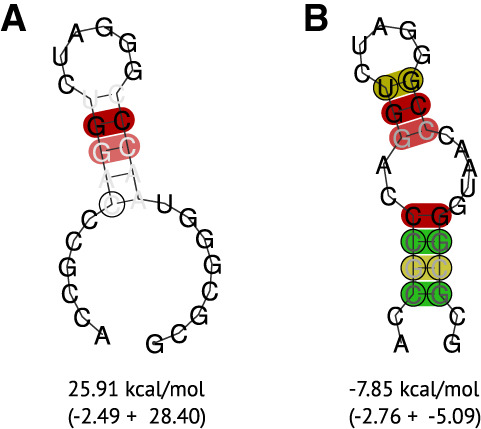
SL IV of species C. The previously reported (*A*) secondary structure (Deng and Brock 1993) can only be formed in four out of 10 representative C sequences (GU233732, KP343640, KC533775, MG387218), whereas our predicted secondary structure of SL IV (*B*) can be formed by all sequences with MFE ranging from −8.10 to −13.50 kcal/mol. The averaged MFE in kcal/mol (composed of actual MFE and covariance pseudo-energy) is displayed below the structure.

Generally, SL IV is present in all sequences of species A; however, it is difficult to reconstruct the evolution of SL IV. Our prediction of SL IV in species C slightly differs from the literature (Fig. 6; Deng and Brock 1993).

SL III has been reported to be AU-rich in strain *C* (Thurner et al. 2004; Coronado et al. 2017). In the present work, we additionally find species D, I, N, O, E, and L to contain such an AU-rich region. Interestingly, species E and L are phylogenetically distant from D, I, N, and O (Fig. 1), and we hypothesize the AU-rich region to have been invented twice during *Pestivirus* evolution. Additionally, species F is phylogenetically related to species E and L, but appears without an AU-rich region.

All other sequences only show similarities within species or some conserved regions at the primary sequence level between some species. The sequences of the species K are very divergent but are generally alignable at a sequence level (Supplemental File F13). Species K and S show fragment-wise sequence similarity. When comparing species K, S, and Zikole, we observe primary sequence similarities in the location of SL III (alignment position 14,151–14,245). Still, we could not predict a conserved secondary structure for all three species. The closely related species Q, J, and R contain the longest sequences within the 3′ UTR, with an insertion upstream of SL I, which shows sequence similarities between the three sequences. The three representative sequences of species P are generally well conserved.

### New RNA secondary structure candidates in the protein-coding region

The importance of RNA secondary structures in the protein-coding region and the underlying selection pressure have been extensively studied, showing that structural conformations in the CDS can be beneficial for viral translation and replication (Pirakitikulr et al. 2016; Fricke et al. 2019). Prediction of RNA secondary structures based on alignments was performed in a previous study for HCV and resulted in over 40 structural elements (including 23 novel ones) in the coding region (Triebel et al. 2024). Although the computational method and overall genome organization are similar here, the results should not be compared because HCV is a species and pestivirus is an entire genus with greater sequence diversity.

**FIGURE 7.**
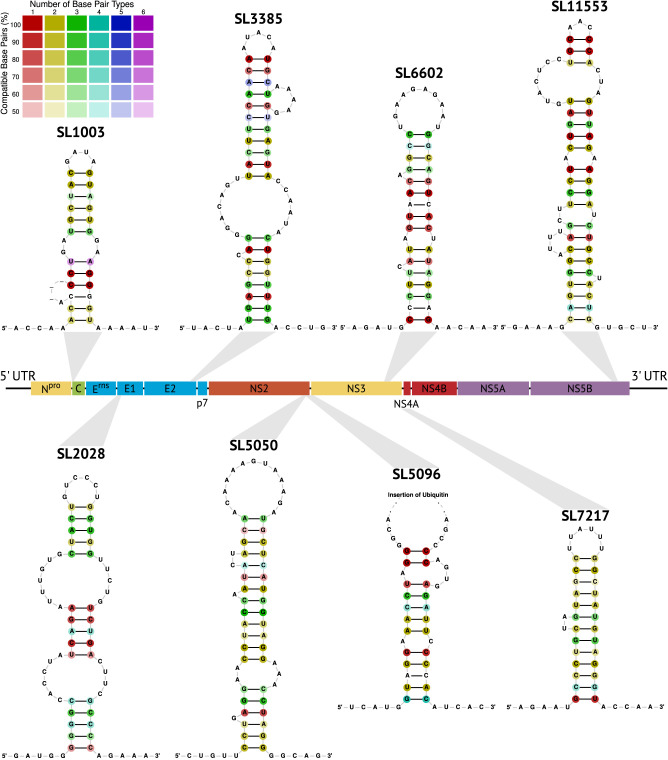
Selection of eight novel conserved RNA secondary structure candidates located in the protein-coding region of the *Pestivirus* alignment. Novel structures are named according to their start position in the genome 1-SD1 (accID NC_076029).

Our analysis identified several novel RNA secondary structures in the protein-coding region using our in silico prediction method (Table 2; Fig. 7; Supplemental Fig. S7). We selected new structure candidates based on the conservation at both the sequence and structural levels, particularly the presence of compensatory mutations. We identified 29 novel structures that are likely functional elements, as they exhibit conserved RNA secondary structures with compensatory mutations despite being located in coding regions (Fig. 7; Supplemental Fig. S7). Furthermore, we identified three structures that are conserved in a subset of species (Table 3).

**TABLE 3.** Conserved RNA secondary structures (SS) in a subset of the *Pestivirus* genus

RNA SS	Genomic region	Alignment position		Genome position		Species (reference genome)
		From	To	From	To	
**SL 637**	*N* ^*pro*^	1186	1224	637	675	A (1-SD1, NC_076029), B, G, H, C, D, I, N, O, E, F, L, Wenzhou
**SL 4652**	*NS2*	5675	5728	4652	4695	A (1-SD1, NC_076029), B, G, H, C, D, I, N, O, E, F, L, M, Wenzhou
**SL 5222**	*NS3*	6863	6889	5222	5248	A (1-SD1, NC_076029), B, G, H, C, D, I, N, O, E, F, L, M, Wenzhou, P, Q, J, R, Jingmen
SL IV (RF04323)	3′ UTR	13,947	14,142	12,114	12,129	A (1-SD1, NC_076029)
SL II (RF04323)	3′ UTR	14,194	14,693	12,141	12,233	A (1-SD1, NC_076029)
SL IV (RF04324)	3′ UTR	13,948	14,117	12,073	12,098	C (Alfort/187, NC_038912)
SL III (RF04324)	3′ UTR	14,119	14,24	12,100	12,174	C (Alfort/187, NC_038912)
SL II (RF04324)	3′ UTR	14,365	14,694	12,175	12,227	C (Alfort/187, NC_038912)
SL III (RF04325)	3′ UTR	14,117	14,245	11,106	11,164	K (NC_038964), S, Zikole

Start and end positions refer to the alignment and reference strain for the corresponding species. We confirmed the previously predicted RNA secondary structures of SL II, SL III, and SL IV in the 3′ UTR for species A and C separately (available in Rfam v15.1 or later) and predicted further three novel conserved RNA families (bold font), which will be incorporated into Rfam. We named novel structures according to their position in the reference genome.

These well-conserved structures represent previously unrecognized RNA elements within representative *Pestivirus* genomes, highlighting the complexity and diversity of RNA secondary structures in this viral genus. The discovery of these novel structures provides new insights into their potential roles in *Pestivirus* replication, translation, and pathogenesis. Further investigations are necessary to experimentally validate their function and explore their biological significance in the context of *Pestivirus* infections.

### Improvements to Rfam virus families

In total, we identified 32 conserved structural regions across the representative genomes of pestiviruses, of which 29 are presented here for the first time. Only the 5′ UTR of the pestiviral genome is currently present as a known RNA secondary structure in Rfam. We improved the IRES model (RF00209) from the Rfam database (Table 2; Griffiths-Jones et al. 2003; Ontiveros-Palacios et al. 2025) to ensure comprehensive coverage of the entire phylogenetic clade of the pestivirus sequences. The previous IRES model contained 25 sequences covering only the species A, B, C, D, and G. We extended the model to 48 sequences covering all 19 species, spanning 508 nt positions in the alignment (previously 286). The new model now includes SL I (from 32 *Pestivirus* genomes), which was absent due to previous sequencing problems of the very 5′ genome end. Importantly, the new model now includes the pseudoknot SL IV.

In addition, we provide alignments for the new models of RNA secondary structures in the 3′ UTR for the Rfam. RF04322 includes SL I, which is conserved throughout the entire genus of pestiviruses (Table 2; Fig. 5), while the models RF04323, RF04324, and RF04325 represent species A, C, and K, respectively (Table 3).

### Genome circularization

**FIGURE 8.**
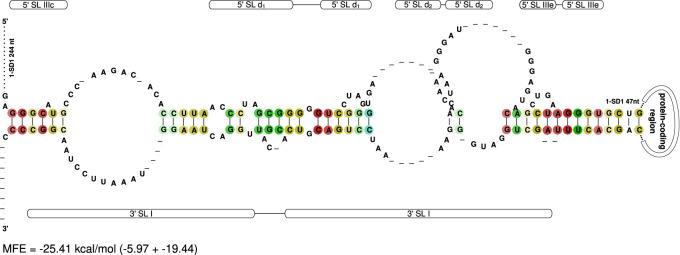
In silico predicted RNA–RNA interaction between the 5′ and 3′ UTRs of 29 representative *Pestivirus* genomes (15 species). The possible genome circularization is located upstream of the start codon and at the very 3′ end of the genome with an MFE of −25.41 kcal/mol.

Genome circularization is a well-known phenomenon and is considered important for viral replication and drug design (Alvarez et al. 2005; Fricke et al. 2015; Huber et al. 2019). In this study, we predicted a potential long-range interaction (LRI) between the 5′ and 3′ UTRs in 29 of our representative *Pestivirus* sequences (15 species), where both UTRs are present (31). Typically, genome circularization involves interactions between the very 5′ end of the 5′ UTR and the 3′ end of the 3′ UTR. However, our prediction suggests that the LRI forms upstream of the polyprotein start codon and the very 3′ end of the 3′ UTR (Fig. 8). This implies that SL IIIc to IIIe in the 5′ UTR and SL I in the 3′ UTR must unfold to allow the LRI to form. While our findings provide new insights into possible genome circularization mechanisms in *Pestivirus*, they remain based solely on in silico predictions and require experimental validation.

Experiments demonstrating that the pestiviral IRES element can be functionally replaced by IRES sequences from HCV or even encephalomyocarditis virus (EMCV) argue at least against an essential role for these interactions (Frolov et al. 1998).

### Larger host insertions

**FIGURE 9.**
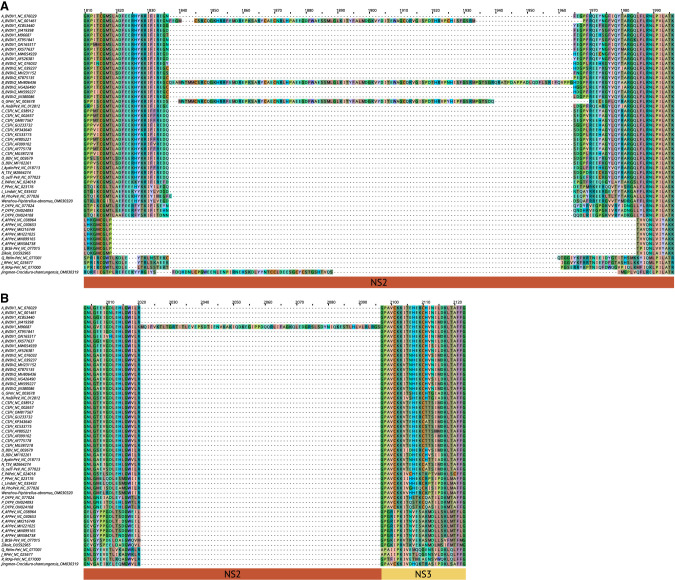
Multiple sequence alignments showing the insertion of (*A*) DNAJC14 (Fig. 2B, orange box) and (*B*) ubiquitin (Fig. 2B, cyan box). (*A*) The insertion is of DNAJC14 present in the NADL strain (species A, accID NC_001461), isolate 296c (species B, accID MH806436), and giraffe-1 H138 (species G, accID NC_003678). An undescribed insertion occurs in the Jingmen isolate SYS_SheQu (species unclassified, accID OM030319). (*B*) The insertion of ubiquitin is only present in the Osloss strain (species A, accID M96687).

Our alignment captures representative pestiviral genomes with insertions from host mRNA, which are reported features of cytopathogenicity. Insertions are typically found in cytopathogenic pestiviruses (Brownlie et al. 1984; Becher et al. 1998, 2002; Becher and Tautz 2011). The insertion of DNAJC14 (nt aln position 6017–6398 and aa aln position 1836–1964) is present in the following three genomes of our representative set (Fig. 2B, orange box; Fig. 9A): strain NADL (species A, accID NC_001461), isolate 296c (species B, accID MH806436), and giraffe-1 H138 (species G, accID NC_003678). DNAJC14 is incorporated into the genome of pestiviruses within the *NS2* gene (see Introduction). The Jingmen isolate SYS_SheQu (species unclassified, accID OM030319) also contains an insertion, which is, however, not homologous to DNAJC14 and also seems not to match any known sequence in the NCBI. Another insertion is known between the genes for *NS2* and *NS3*. This is the ubiquitin insertion (nt aln position 6563–6793 and aa aln position 2021–2095), which is only present in one of our representatives: the Osloss strain (species A, accID M96687) (Fig. 2B, cyan box; Fig. 9B). Other insertions, such as NEDD3, SUMO, and LC3 (Becher and Tautz 2011), are not included in the MSA, as these sequences are only part of partial genomes and therefore do not occur in our original data set. We expect that extensive sequencing in the next few years will lead to genome sequences with insertions that can be included in our alignment. Our results show that our method can preferentially select representative genomes that are divergent in terms of insertions.

## CONCLUSION

In this study, we present the first comprehensive genome-wide multiple sequence alignment (MSA) of pestivirus genomes, incorporating computational predictions of RNA secondary structures. Our approach leveraged a representative selection of 55 genomes, chosen through HDBSCAN clustering of *k*-mer distributions and dimension reduction, effectively capturing the phylogenetic and functional diversity of the genus, for example, host mRNA insertions. The alignment integrates existing genome annotations, conserved structural elements, and pseudoknots, facilitating seamless comparison with other research studies. By incorporating suboptimal structure predictions via tools such as LocARNA and RNAalifold, we reveal potential alternative RNA conformations that may play regulatory roles. These findings expand our understanding and provide new insights into the viral “life cycle” of pestiviruses.

While our study offers valuable insights into conserved RNA secondary structures in pestivirus genomes, it has limitations. It does not automatically account for long-range RNA–RNA interactions across the whole genome, three-dimensional RNA conformations, or RNA–protein interactions, all of which may play critical regulatory roles and warrant further investigation. The computational challenges associated with modeling such long-range interactions remain unresolved and have been tackled only partially in the past, as for HCV (Fricke and Marz 2016). In addition, future studies should aim to validate the predicted structures and their biological relevance in vivo, identify other potentially involved miRNAs, and assess the functional implications of these conserved elements.

All conserved RNA secondary structure models identified here will be included in Rfam version 15.1 or later, ensuring broad accessibility and utility. This alignment provides a standardized and extensible framework for future pestivirus studies, serving as a foundational resource for comparative genomics, evolutionary analyses, and functional investigations.

## MATERIALS AND METHODS

### Data

We downloaded a total of 1684 *Pestivirus* genomes (October 11, 2023) from the BV-BRC and NCBI databases (Sayers et al. 2022; BV-BRC 2023). To ensure the quality of the data, we filtered the genome status “complete” and excluded the host group “lab”.

After identifying redundant genomes by utilizing the preprocessing step of our ViralClust (S Triebel, K Lamkiewicz, and M Marz, unpubl.) pipeline, the data set was refined to a total of 756 genomes. Both the original data set and the prefiltered data set are included in Supplemental Files F1 and F2 in .fasta format.

### Selection of representative genomes

We clustered the prefiltered data set based on *k*-mers to select sequences representing the entire data set: First, we calculated the *k*-mer profiles of each input sequence. Then, we performed a dimension reduction by principal component analysis (PCA), implemented in scikit-learn (v1.2.2) (Pedregosa et al. 2011), followed by clustering using HDBSCAN v0.8.27 (McInnes et al. 2017). We calculated centroid sequences based on the average pairwise distance between all vectors in the same cluster. The sequence with the minimum average distance is selected as the “representative genome” for this cluster. The resulting 52 representative genomes are well distributed throughout the known *Pestivirus* genomes. For comparison, we additionally clustered sequences with these four algorithms: cd-hit-est (Li and Godzik 2006; Fu et al. 2012), MMSeqs2 (Steinegger and Söding 2017), sumaclust (Mercier et al. 2013), and vclust (Rognes et al. 2016). The workflow is implemented in ViralClust (S Triebel, K Lamkiewicz, and M Marz, unpubl). Based on these results (Supplemental Table S1), we only used HDBSCAN for further analysis. We manually edited our set of representative genomes by (a) adding all 23 RefSeq genomes from NCBI (Sayers et al. 2022) and ICTV (Supplemental Table S2 “RefSeqs”; Lefkowitz et al. 2018; Walker et al. 2020), (b) adding seven genomes representing outliers or subtrees not covered by the clustering results (Supplemental Table S2 “Outlier”), (c) reducing 23 overrepresented genomes from species A, B, C, and K (Supplemental Table S2 “Reduce overrepresentation”), and (d) removed one genome (JQ799141) due to a truncated protein. The representative genomes and their distribution throughout the pestiviruses are highlighted in Figure 1. We visualized the nonredundant data set in a split graph using MAFFT (Katoh et al. 2002) and subsequently SplitsTree (Bryant 2003; Huson and Bryant 2006). The phylogenetic tree is stored in Supplemental File F3. For the generation of the alignment presented here, we removed the three Pesti-like viruses (ON684360, MN025505, MW256672), since the sequences are over 4000 nt longer (16,232, 16,274, and 16,802 nt) than the average of the other *Pestivirus* sequences (∼12,200 nt) and they show a high phylogenetic distance in the split graph (Fig. 1). A total of 55 representative genomes covering all 19 species of pestiviruses were selected (Supplemental File F4).

### Multiple sequence alignment and RNA secondary structure prediction

The final set of 55 representative genomes served as input for constructing the full-genome multiple sequence alignment (MSA), including RNA secondary structure information for the entire sequence. We generated this alignment in five steps by cutting it into subalignments, processing, and concatenating them: (1) We identified highly conserved regions (called “anchors”) using AnchoRNA v1.0.1 (Supplemental File F5; Eulenfeld et al. 2025). Anchors are regions in the translated amino acid sequences present in all genomes, requiring a minimum length of 5 and a minimum BLOSUM62 score of 22 between each anchor in the anchor region and at least one other anchor in the same anchor region. For a detailed description of AnchoRNA itself and its potential to detect primer sites, we refer to Supplemental Table S3, Supplemental Figure S8, and Eulenfeld et al. (2025). (2) Subsequently, we focused our analysis on the subregions between these anchors, as well as the 5′ UTR and the 3′ UTR, using LocARNA v2.0.0 with additional parameters ‐‐stockholm ‐‐consensus-structure alifold (Will et al. 2007). We temporarily removed for this step 25 highly similar sequences (Supplemental Table S2 “Highly similar sequences”) to bring less bias into the covariance model calculation. (3) The subalignments with the predicted RNA secondary structures were manually curated. At the end of this step, we added the temporarily removed 25 highly similar sequences again to the alignment. (4) We merged the subalignments according to the anchors into one overall MSA. We modified the sequence of ON165517 in two residues to remove a frameshift in the alignment: (a) changed “-” to “N” in alignment position 3371 and (b) deleted “A” in sequence position 2672. (5) We searched for RNA secondary structures using a sliding window approach (RNALalifold) and analyzed genome circularization using the ViennaRNA package (Lorenz et al. 2011). (6) The coding sequence of the nucleotide alignment is then translated into the amino acid alignment.

After we had built the full-genome alignment with RNA secondary structure information, we added the annotation of proteins and conserved RNA secondary structures described in the literature. Finally, we selected highly conserved RNA secondary structure regions to export to the Rfam database (Griffiths-Jones et al. 2003; Ontiveros-Palacios et al. 2025).

### Visualization

Alignments were visualized using Jalview (Clamp et al. 2004). In the case of protein alignments, we used the gecos Ocean coloring (based on BLOSUM62) (Kunzmann et al. 2020). The overview alignment was visualized using sugar (Eulenfeld 2025). The alignment-based structures were visualized using R2DT (Sweeney et al. 2021) for the 2D representation and RNAalifold (Lorenz et al. 2011) for coloring. Single-sequence RNA secondary structure predictions were done using RNAfold with parameter -p for partition function. If stated, an additional parameter for structural constraints (-C) is used (Lorenz et al. 2011).

## DATA DEPOSITION

Data are available in the Supplemental Material and via Zenodo https://doi.org/10.5281/zenodo.15490752. The RNA secondary structure models are provided in the Rfam database (Griffiths-Jones et al. 2003; Ontiveros-Palacios et al. 2025).

## CODE AVAILABILITY

ViralClust is available via GitHub https://github.com/rnajena/viralclust (S Triebel, K Lamkiewicz, and M Marz, unpubl.). AnchoRNA is available via GitHub https://github.com/rnajena/anchorna (Eulenfeld et al. 2025). Sugar is available via GitHub https://github.com/rnajena/sugar (Eulenfeld 2025).

## SUPPLEMENTAL MATERIAL

Supplemental material is available for this article.

## Figures and Tables

**Sandra Triebel:**
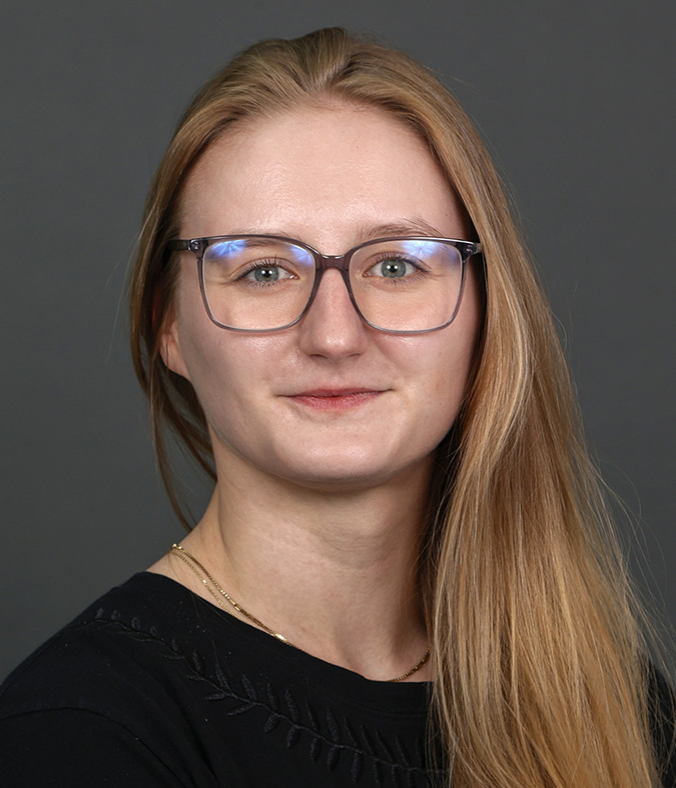


## MEET THE FIRST AUTHOR

***Meet the First Author(s)* is an editorial feature within *RNA*, in which the first author(s) of research-based papers in each issue have the opportunity to introduce themselves and their work to readers of *RNA* and the RNA research community. Sandra Triebel is the first author of this paper, “First full-genome alignment representative for the genus *Pestivirus*.” Sandra is a PhD student in the group for RNA Bioinformatics & High-Throughput Analysis at the Friedrich Schiller University Jena, Germany. Her research focuses on RNA secondary structures in viral genomes and multiple sequence alignments of complete genomes.**

**What are the major results described in your paper, and how do they impact this branch of the field?**

In this work, we present the first representative full-genome alignment for the entire genus *Pestivirus*. Our study integrates RNA secondary structure prediction across the full genomic diversity of the genus, providing a comprehensive resource for both sequence- and structure-based analyses. We confirm multiple previously described and experimentally validated RNA structures and, importantly, uncover several novel conserved structural elements across pestivirus genomes. This alignment provides a foundation for comparative studies, functional RNA structure analyses, and future investigations into *Pestivirus* evolution, replication, and pathogenicity.

**What led you to study RNA or this aspect of RNA science?**

Viruses have always fascinated me because they are remarkably efficient at using extremely compact genomes to control complex biological processes. RNA secondary structures play a central role in this efficiency, and I find it particularly exciting that the conservation of RNA structure often extends far beyond conservation of the primary sequence itself. Understanding how structure, rather than sequence alone, is preserved across evolution continues to inspire my interest in RNA biology and viral genomics.

**During the course of these experiments, were there any surprising results or particular difficulties that altered your thinking and subsequent focus?**

Generating a full-genome alignment for an entire viral genus posed substantial challenges. The high sequence diversity of pestiviruses, together with their ability to incorporate host mRNA fragments into their genomes, complicated conventional alignment strategies. These difficulties ultimately led us to adopt a divide-and-conquer approach for alignment generation, which proved essential for handling the complexity of the data and achieving a meaningful, structure-informed consensus.

**What are some of the landmark moments that provoked your interest in science or your development as a scientist?**

There was no single defining moment that sparked my interest in science. Rather, I have always been deeply impressed by what can be achieved when curious and creative minds collaborate as a team. Being part of such collaborative efforts and contributing to the discovery of something genuinely new continues to be one of the most motivating aspects of my work as a scientist.

**If you were able to give one piece of advice to your younger self, what would that be?**

I would advise my younger self to be braver and have greater confidence in my own abilities. Trusting yourself and being willing to take risks are essential components of personal and scientific growth.
